# Propofol Attenuates Hypoxia-Induced Inflammation in BV2 Microglia by Inhibiting Oxidative Stress and NF-*κ*B/Hif-1*α* Signaling

**DOI:** 10.1155/2020/8978704

**Published:** 2020-04-28

**Authors:** Xiaowei Peng, Chenglong Li, Wei Yu, Shuai Liu, Yushuang Cong, Guibo Fan, Sihua Qi

**Affiliations:** Department of Anesthesiology, The Fourth Affiliated Hospital of the Harbin Medical University, 37 Yiyuan Road, Harbin, 150001 Heilongjiang, China

## Abstract

Hypoxia-induced neuroinflammation typically causes neurological damage and can occur during stroke, neonatal hypoxic-ischemic encephalopathy, and other diseases. Propofol is widely used as an intravenous anesthetic. Studies have shown that propofol has antineuroinflammatory effect. However, the underlying mechanism remains to be fully elucidated. Thus, we aimed to investigate the beneficial effects of propofol against hypoxia-induced neuroinflammation and elucidated its potential cellular and biochemical mechanisms of action. In this study, we chose cobalt chloride (CoCl_2_) to establish a hypoxic model. We found that propofol decreased hypoxia-induced proinflammatory cytokines (TNF*α*, IL-1*β*, and IL-6) in BV2 microglia, significantly suppressed the excessive production of reactive oxygen species, and increased the total antioxidant capacity and superoxide dismutase activity. Furthermore, propofol attenuated the hypoxia-induced decrease in mitochondrial membrane potential andy 2 strongly inhibited protein expression of nuclear factor-kappa B (NF-*κ*B) subunit p65 and hypoxia inducible factor-1*α* (Hif-1*α*) in hypoxic BV2 cells. To investigate the role of NF-*κ*B p65, specific small interfering RNA (siRNA) against NF-*κ*B p65 were transfected into BV2 cells, followed by exposure to hypoxia for 24 h. Hypoxia-induced Hif-1*α* production was downregulated after NF-*κ*B p65 silencing. Further, propofol suppressed Hif-1*α* expression by inhibiting the upregulation of NF-*κ*B p65 after exposure to hypoxia in BV2 microglia. In summary, propofol attenuates hypoxia-induced neuroinflammation, at least in part by inhibiting oxidative stress and NF-*κ*B/Hif-1*α* signaling.

## 1. Introduction

Microglia are the primary neuroimmune cells important for surveillance and defense, in addition to maintaining homeostasis, being the first sensors of pathophysiological changes, and triggering subsequent cascade reactions [1–3]. Microglia undergo a remarkable transformation into “activated microglia” during brain injury and disease, when numerous genes are switched on. M1-activated microglia mainly secrete proinflammatory cytokines (TNF-*α*, IL-1*β*, and IL-6), which consequently promote the development of several central nervous system (CNS) disorders. However, M2-activated microglia primarily secrete anti-inflammatory cytokines, which can facilitate tissue reconstruction [4, 5]. In a hypoxic condition, Hif-1*α* is stabilized and activates the transcription of genes associated with proinflammation [6, 7]. Moreover, hypoxia impairs mitochondrial function resulting in increased production of reactive oxygen species (ROS), decreased mitochondrial membrane potential, and decreased ATP production [8, 9]. Considering the key role of neuroinflammation in hypoxia-induced nerve damage, neuroinflammation induced by activated microglia (especially M1 polarization) is an important regulator of hypoxia-induced CNS damage.

As a commonly used intravenous anesthetic, propofol has many other functions [10, 11]. In our previous study, we found that propofol can prevent oxidative stress and attenuate mitochondrial dysfunction during focal cerebral ischemia-reperfusion injury [12, 13]. Further, propofol has an anti-inflammatory effect as it inhibits NF-*κ*B activation in mice with allergic asthma [14]. NF-*κ*B subunit p65 has been reported to induce basal level expression of Hif-1*α* mRNA and protein [15–17]. However, the mechanism by which propofol attenuates hypoxia-induced neuroinflammation in activated microglia is relatively unclear. Therefore, we designed this study to investigate the protective effect of propofol on hypoxia-induced neuroinflammation associated with microglia and whether this occurs through the inhibition of oxidative stress and NF-*κ*B/Hif-1*α* signaling.

## 2. Materials and Methods

### 2.1. Microglial Cell Culture

Since BV2 microglia originate from mouse brains, they share several phenotypic characteristics with primary microglia. Thus, we chose these cells as the model for the current study. The cells were obtained from the National Infrastructure of Cell Line Resource (China). Microglia were grown according to the recommended conditions described by the cell bank. The medium contained high-glucose Dulbecco's modified Eagle's medium (DMEM) (Corning, USA) with 10% fetal bovine serum (FBS) (Corning, USA) and 1% penicillin-streptomycin. The cells were incubated at 37°C in a humidified atmosphere of 95% air and 5% CO_2_. After growing them to 80% confluence, cells were subcultured two or three times every week. Microglia were seeded in a 6-well plate for 24 h for subsequent experiments, and the inoculation density was 0.5 × 10^6^ cells/ml.

### 2.2. Cell Treatment

In this study, 300 *μ*M CoCl_2_ was used to establish hypoxia. According to the preliminary results of cell viability, the 50% inhibitive concentration (IC50) of CoCl_2_ was 300 *μ*M at 24 h. Moreover, Hif-1*α* protein was expressed stably. CoCl_2_ was dissolved in distilled water to 300 mM. CoCl_2_ solution was filtered with a 0.22 mm Millipore membrane and stored at -20°C. Serum-free medium was used to dilute CoCl_2_ for cell treatment. Propofol was used to pretreat cells for 3 h before CoCl_2_ treatment. Following propofol treatment, the cells were washed twice with PBS.

### 2.3. Real-Time Quantitative PCR (qPCR)

Microglia were treated as indicated, and total RNA was collected with a TRIzol reagent (Sigma, America). cDNA was prepared using 1 *μ*g of total RNA as the template with a reverse transcription kit (Takara, Japan). The reverse transcription apparatus was set up according to the kit instructions. Real-time PCR analysis was performed using an ABI Prism 7500 fast real-time PCR System (Applied Biosystems, America) with SYBR green. *ΔΔ*CT values were used for analysis. qPCR was set up with a SYBR green PCR master mix (Roche, Switzerland), specific primers, cDNA, and double-distilled water. The conditions for PCR cycles were as follows: predenaturation (94°C for 2 min), denaturation (94°C for 15 s), annealing, and extension (60°C for 30 s). *β*-Actin was used as a housekeeper gene control, and untreated cells were used as a control to normalize the relative amounts of target gene expression. qPCR primer sequences are shown in Table 1.

### 2.4. Enzyme-Linked Immunosorbent Assay (ELISA)

Microglia were treated as indicated, and cell supernatants were used to measure the concentration of TNF-*α*, IL-1*β*, and IL-6 with ELISA kits (BOSTER, China), according to the manufacturer's instructions. The absorbance, using an iMark microplate reader (SpectraMax M3, Molecular Devices, America), was measured at a wavelength of 450 nm. The cytokine concentrations were determined based on reference standard curves.

### 2.5. Western Blotting

After treating microglia as indicated, cells were lysed with RIPA buffer containing a protease inhibitor and phosphatase inhibitor (BOSTER, China) at a ratio of 100 : 1 : 1. After the cells were completely lysed, the supernatant was taken, and the protein concentration was measured using a BCA protein quantitative kit (BOSTER, China). The same amount of protein was separated by sodium dodecyl sulfate/polyacrylamide gel electrophoresis (SDS-PAGE) and transferred to a polyvinylidene fluoride (PVDF) membrane (Roche, Switzerland). Tween 20 (BOSTER, China) in Tris-buffered saline (BOSTER, China), containing 5% skim milk powder, was used to block the membrane for 2 h. Primary antibodies against NF-*κ*B p65 (1 : 1400, ab16502, Abcam), Hif-1*α* (1 : 1000, 14179, Cell Signaling Technology), and GAPDH (1 : 1000, TA-08, ZSGB-Bio) were then incubated with PVDF membranes at 4°C overnight. Next, secondary antibodies (peroxidase-labeled) were incubated with the blots at 25°C for 1 h. The protein bands were observed using the enhanced chemiluminescence (ECL) system (GE Healthcare, UK), and relative protein expression was quantified using ImageJ software.

### 2.6. Reactive Oxygen Species Assay Using Flow Cytometry

Drug intervention was completed as expected, and cells were stained with DCFH-DA (10 *μ*M) for 20 min at 37°C in the dark. Following this, the cells were washed twice with PBS. Cells were removed from the plate surface and transferred to polypropylene FACS tubes with 500 *μ*l PBS. Finally, a MoFlo XDP flow cytometer (Beckman, USA) was used for measurements and FlowJo software for data analysis.

### 2.7. Observation of Mitochondrial Membrane Potential Using Confocal Laser Scanning Microscopy

BV-2 cells were first seeded in confocal dishes. After treatment with different drugs, cells were stained with TMRM (20 nM, T668, Thermo Fisher Scientific) for 30 min at 37°C in the dark. The cells were then washed twice with PBS. Subsequently, the fluorescence intensity was measured by confocal laser scanning microscopy at an excitation wavelength of 561 nm, and data were analyzed using ImageJ software.

### 2.8. NF-*κ*B p65 siRNA Transfections

siRNA targeted at NF-*κ*B p65 (Sangon Biotech, China) was used to knock down NF-*κ*B p65. The target sequence was 5′-CAACCATGGCTGAAGGAAA-3′. First, 100 pmol NF-*κ*B p65 siRNA duplex was diluted into 250 *μ*l Opti-MEM medium. Second, 5 *μ*l lipofectamine 2000 (Lipo2000) transfection reagent (Thermo Fisher Scientific, America) was diluted with 250 *μ*l Opti-MEM medium and incubated for 5 min at room temperature in a separate tube. Third, the aforementioned solutions were mixed gently and incubated for 30 min at 25°C to form a transfection complex. Next, 70% confluent BV2 cells to be used in the study were washed thrice with PBS. Following this, 1500 *μ*l Opti-MEM medium and 500 *μ*l transfection complex were added. The cells were incubated for 24 h at 37°C. Western blotting was performed to detect NF-*κ*B p65 protein levels.

### 2.9. Statistical Analysis

Statistical analyses were performed using IBM SPSS Statistics 24 software. All data presented are representative of at least three independent experiments. The data are shown as the mean ± standard errors of the mean (S.E.M.). Independent Student's *t*-test was used for comparisons between two groups, and one-way analysis of variance (ANOVA) with the SNK post hoc test was used to compare multiple groups. The result was considered to be statistically significant when the *P* value was less than 0.05.

## 3. Results

### 3.1. CoCl_2_ Exposure Induces the Expression of Proinflammatory Cytokines in BV2 Cells

**Figure 1:**
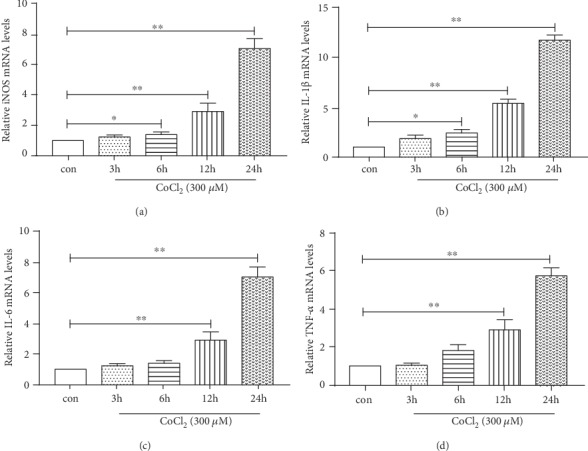
CoCl_2_ induces *iNOS*, *IL-1β*, *IL-6*, and *TNF-α* mRNA expression in microglia. BV2 cells were treated with 300 *μ*M of CoCl_2_ for the indicated times (3, 6, 12, and 24 h). *iNOS*, *IL-1β*, *IL-6*, and *TNF-α* mRNA expression was detected by qPCR. The relative amounts of transcripts were calculated using the 2^−*ΔΔ*CT^ formula. *β*-Actin mRNA was used as the internal control. (a) *iNOS* mRNA expression at different times. (b) *IL-1β* mRNA expression at different times. (c) *IL-6* mRNA expression at different times. (d) *TNF-α* mRNA expression at different times. Data from at least three independent experiments are expressed as the mean ± S.E.M. ANOVA with the SNK post hoc test was used to assess differences between groups. ^∗^*P* < 0.05, ^∗∗^*P* < 0.01 versus the control group.

To observe the effect of CoCl_2_ treatment time on inflammatory responses in microglia, BV2 cells were treated with CoCl_2_ (300 *μ*M) for 3, 6, 12, and 24 h. *TNF-α*, *IL-1β*, *IL-6*, and *iNOS* mRNA expression was detected by qPCR. As shown in Figure 1, *TNF-α*, *IL-1β*, *IL-6*, and *iNOS* mRNA expression was significantly increased in CoCl_2_-treated cells at 12 and 24 h compared to that in control cells (^∗∗^*P* < 0.01). Further, compared to that in control cells, *IL-1β* and *iNOS* expression was significantly increased in cells treated with CoCl_2_ at 6 h (^∗^*P* < 0.05). Based on the results, we infer that the expression of cytokines in BV2 cells was remarkably increased with prolonged CoCl_2_ incubation times.

### 3.2. Propofol Inhibits CoCl_2_-Induced Production of Proinflammatory Cytokines

**Figure 2:**
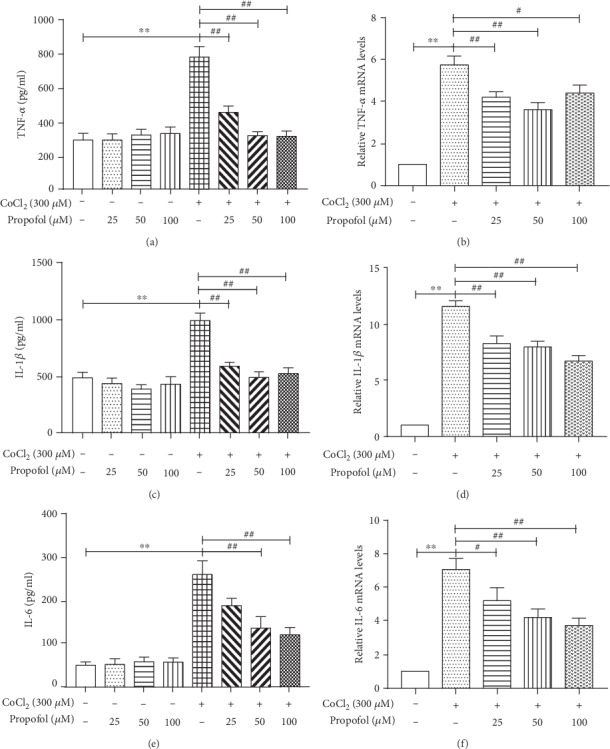
Propofol reduces the secretion of TNF-*α*, IL-1*β*, and IL-6 in CoCl_2_-treated microglia. BV2 cells were pretreated with propofol (25, 50, and 100 *μ*M) for 3 h, which was followed by treatment with CoCl_2_ (300 *μ*M) for 24 h. IL-1*β*, TNF-*α*, and IL-6 were analyzed by ELISA and qPCR. (a) *TNF-α* concentration after different treatments. (b) Relative *TNF-α* mRNA levels after different treatments. (c) *IL-1β* concentration after different treatments. (d) Relative *IL-1β* mRNA levels after different treatments. (e) *IL-6* concentration after different treatments. (f) Relative *IL-6* mRNA levels after different treatments. Data from at least three independent experiments are expressed as the mean ± S.E.M. Independent Student's *t*-test was used for comparisons between two groups, and ANOVA with the SNK post hoc test was used for multiple groups. ^∗∗^*P* < 0.01 versus the control group; ^##^*P* < 0.01 versus the CoCl_2_ group.

To determine whether propofol has an effect on proinflammatory cytokines, we pretreated cells with different concentrations of propofol (25, 50, and 100 *μ*M) for 3 h before CoCl_2_ stimulation for 24 h and then measured *TNF-α*, *IL-1β*, and *IL-6* protein and mRNA levels. As displayed in Figure 2, CoCl_2_ increased both protein and mRNA levels of *TNF-α*, *IL-1β*, and *IL-6* compared to control cells. Propofol inhibited protein secretion of *TNF-α* and *IL-1β* at 25, 50, and 100 *μ*M concentrations; however, protein secretion of *IL-6* was inhibited at 50 and 100 *μ*M propofol concentrations only. Furthermore, propofol reduced mRNA levels of *TNF-α*, *IL-1β*, and *IL-6* at 25, 50, and 100 *μ*M concentrations. To summarize, propofol could reduce CoCl_2_-induced proinflammatory cytokine levels in BV2 cells, but not in a concentration-dependent manner.

### 3.3. Propofol Ameliorates CoCl_2_-Induced Oxidative Stress

**Figure 3:**
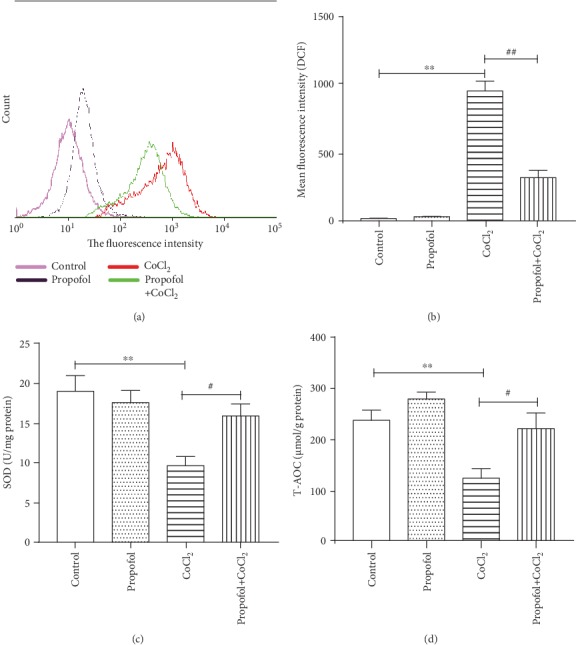
Propofol ameliorates CoCl_2_-induced oxidative stress in microglia. BV2 cells were pretreated with propofol (50 *μ*M) for 3 h, followed by treatment with CoCl_2_ (300 *μ*M) for 24 h. ROS were analyzed by flow cytometry. SOD and T-AOC were measured using specific kits. (a) ROS levels detected by flow cytometry. (b) The mean fluorescence intensity of DCF staining reflects ROS levels. (c) SOD levels after different treatments. (d) T-AOC levels after different treatments. Data from at least three independent experiments are expressed as the mean ± S.E.M. Independent Student's *t*-test was used for comparisons between two groups. ^∗∗^*P* < 0.01 versus the control group; ^#^*P* < 0.05 versus the CoCl_2_ group; ^##^*P* < 0.01 versus the CoCl_2_ group.

To observe the effect of propofol on CoCl_2_-induced oxidative stress, cells were pretreated with propofol for 3 h before CoCl_2_ stimulation for 24 h. Subsequently, ROS, superoxide dismutase (SOD) activity, and total antioxidant capacity (T-AOC) were detected. As shown in Figures 3(a) and 3(b), CoCl_2_ induced higher ROS production in BV2 cells compared to control cells, whereas propofol decreased ROS levels compared to CoCl_2_-treated cells. As displayed in Figures 3(c) and 3(d), CoCl_2_ decreased SOD and T-AOC activity compared to that in control cells, but propofol ameliorated this reduction. In summary, propofol could ameliorate CoCl_2_-induced oxidative stress in BV2 cells.

### 3.4. Propofol Ameliorates the Decrease in Mitochondrial Membrane Potential in CoCl_2_-Treated Microglia

**Figure 4:**
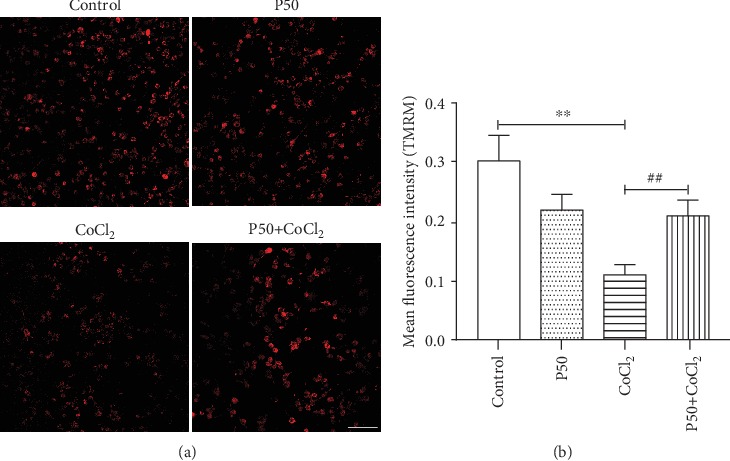
Propofol ameliorates the decrease in mitochondrial membrane potential in CoCl_2_-treated microglia. BV2 cells were pretreated with propofol (50 *μ*M) for 3 h followed by treatment with CoCl_2_ (300 *μ*M) for 24 h. Confocal laser scanning microscopy was used to observe mitochondrial membrane potential. Scale bar: 100 *μ*m. (a) TMRM staining observed by confocal laser scanning microscopy. (b) The mean fluorescence intensity of TMRM staining reflects the membrane potential. The average fluorescence value is shown as the mean ± S.E.M. Data are based on at least three independent experiments. Independent Student's *t*-test was used for comparisons between two groups. ^∗∗^*P* < 0.01 versus the control group, ^#^*P* < 0.05 versus the CoCl_2_ group. p50: propofol 50 *μ*M.

To determine whether propofol affects the CoCl_2_-induced decrease in mitochondrial membrane potential in BV2 cells, we pretreated cells with propofol for 3 h before CoCl_2_ stimulation for 24 h. We then measured mitochondrial membrane potential by confocal laser scanning microscopy. As shown in Figure 4, CoCl_2_ induced a decrease in mitochondrial membrane potential in BV2 cells compared to control cells, whereas propofol attenuated this decrease.

### 3.5. Propofol Attenuates Hypoxia-Induced Neuroinflammation via NF-*κ*B/Hif-1*α* Signaling

**Figure 5:**
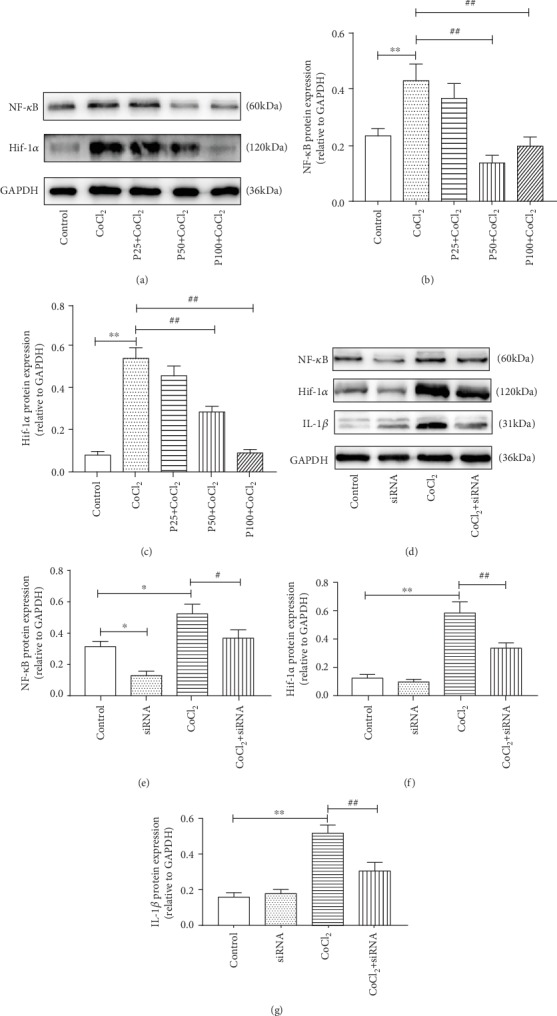
Propofol suppresses NF-*κ*B/Hif-1*α* production in CoCl_2_-treated BV2 cells. BV2 cells were pretreated with propofol (25, 50, and 100 *μ*M) for 3 h followed by treatment with CoCl_2_ for 24 h, and cells were examined for NF-*κ*B p65, Hif-1*α*, and IL-1*β* production by western blotting (a–c). (a) The protein levels of NF-*κ*B and Hif-1*α*. (b) The gray value for NF-*κ*B is displayed as a percentage of GAPDH protein levels. (c) The gray value for Hif-1*α* is displayed as a percentage of GAPDH protein levels. We first pretreated cells with siRNA specific for NF-*κ*B p65 for 24 h, followed by CoCl_2_ (300 *μ*M) treatment for 24 h, and then examined NF-*κ*B p65, Hif-1*α*, and IL-1*β* production by western blotting (d–g). (d) The protein levels of NF-*κ*B p65, Hif-1*α*, and IL-1*β*. (e) The gray value for NF-*κ*B is displayed as a percentage of GAPDH protein levels. (f) The gray value for Hif-1*α* is displayed as a percentage of GAPDH protein levels. (g) The gray value for IL-1*β* is displayed as a percentage of GAPDH protein levels. Gray values are shown as the mean ± S.E.M. Data are based on at least three independent experiments. Independent Student's *t*-test was used for comparisons between two groups, and ANOVA with the SNK post hoc test was used for multiple groups. ^∗^*P* < 0.05 versus the control group; ^∗∗^*P* < 0.01 versus the control group; ^#^*P* < 0.05 versus the CoCl_2_ group; ^##^*P* < 0.01 versus the CoCl_2_ group.

To examine the mechanism by which propofol decreased CoCl_2_-induced secretion of proinflammatory cytokines in BV2 cells, we investigated two proteins that propofol could potentially affect within the cell, namely, NF-*κ*B p65 and Hif-1*α*. First, we pretreated cells with different concentrations of propofol (25, 50, and 100 *μ*M) for 3 h before CoCl_2_ stimulation for 24 h and then examined NF-*κ*B p65 and Hif-1*α* production by western blotting. As illustrated in Figures 5(a)–5(c), compared to the CoCl_2_-treated group, propofol significantly suppressed NF-*κ*B p65 and Hif-1*α* production (*P* < 0.05). To further investigate the intrinsic mechanism with respect to NF-*κ*B p65, siRNA against NF-*κ*B p65 were transfected into BV2 cells and incubated for 24 h, followed by exposure to hypoxia for 24 h. As shown in Figures 5(d), 5(f), and 5(g), Hif-1*α* and IL-1*β* were downregulated in NF-*κ*B p65-silenced and CoCl_2_-treated cells compared to that in only CoCl_2_-treated cells (*P* < 0.05). To summarize, propofol suppressed Hif-1*α* production by inhibiting the upregulation of NF-*κ*B p65 in CoCl_2_-treated BV2 microglia. Propofol attenuated hypoxia-induced inflammation in BV2 cells, at least in part by NF-*κ*B/Hif-1*α* signaling.

## 4. Discussion

Propofol, widely used as a short-acting intravenous anesthetic, has chemical properties similar to those of tocopherol, a phenolic free radical scavenger. Moreover, it is lipophilic and can quickly enter cells and subcellular membrane compartments. Thus, propofol has many other functions besides its anesthetic effect [18, 19]. In our previous study, we found that propofol can prevent oxidative stress and attenuate mitochondrial dysfunction upon focal cerebral ischemia-reperfusion injury [12, 13]. Furthermore, it has an antineuroinflammatory effect and can also inhibit NF-*κ*B activation [20]. NF-*κ*B subunit p65 was reported to contribute to basal levels of Hif-1*α* mRNA and protein expression [15–17, 21]. During hypoxia, Hif-1*α* is stabilized and activates the transcription of genes associated with proinflammation [6, 7]. However, the protective effect of propofol against hypoxia-induced neuroinflammation has rarely been reported. In this study, we found that propofol has a protective effect against hypoxia-induced neuroinflammation, at least in part by inhibiting oxidative stress and NF-*κ*B/Hif-1*α* signaling.

Microglia are the primary neuroimmune cells participating in surveillance and defense, first sensing pathophysiological changes in the brain, and triggering subsequent cascade reaction [1–3, 22]. Hypoxia contributes to many central systemic diseases, such as stroke, neonatal hypoxic-ischemic encephalopathy, Alzheimer's disease, Parkinson's disease, vascular dementia, and epilepsy [23–26]. Hypoxia leads to activation of M1 microglia, which mainly secrete neurotoxic substances such as proinflammatory cytokines (*TNF-α*, *IL-1β*, and *IL-6*) and ROS [27, 28]. Proinflammatory cytokines and ROS can directly damage neurons and trigger subsequent cascade reactions. Therefore, inhibiting hypoxia-induced proinflammatory cytokine production is the key to regulating the neuroinflammatory response after hypoxia.

During hypoxia, Hif-1*α* is stabilized and binds to Hif-1*β* and subsequently migrates to the nucleus where it activates transcription of genes associated with inflammation and oxidative stress, causing tissue damage [6, 7, 29, 30]. In this study, we chose CoCl_2_ treatment to mimic hypoxia. CoCl_2_ is known as a hypoxia-mimetic agent in cell line models, as it induces biochemical and molecular reactions similar to those observed under hypoxic conditions [31–34]. In our study, we observed that with prolonged incubation with CoCl_2_, the expression of *TNF-α*, *IL-1β*, *IL-6*, and *iNOS* significantly increased in BV2 cells (Figure 1). As reported [35], Hif-1*α* was also markedly upregulated in hypoxia-induced BV2 cells compared to normoxic cells (Figures 5(a) and 5(d)). It turns out that CoCl_2_-induced inflammation in BV2 cells is associated with Hif-1*α*. Moreover, proinflammatory cytokines (TNF*α*, IL-1*β*, and IL-6) were decreased in hypoxic BV2 cells pretreating with propofol (Figure 2). Propofol also inhibited Hif-1*α* protein expression in hypoxic BV2 cells (Figures 5(a) and 5(c)).

It is known that hypoxia impairs mitochondrial function resulting in ROS production, decreased mitochondrial membrane potential, and metabolic changes [8, 9]. ROS are proinflammatory factors which initiate inflammatory cascade reactions and are the main signaling molecules that regulate macrophage phagocytosis and killing [8, 36]. ROS oxidizes Fe^2+^ to Fe^3+^, an important cofactor that inhibits prolyl hydroxylase activity, and indirectly stabilizes Hif-1*α* protein [37]. However, overproduction of ROS can lead to oxidative stress, resulting in decreased SOD and T-AOC activity. SOD can also inhibit the activation of NF-*κ*B to limit the inflammatory response [38]. Further, oxidative stress amplifies microglia inflammatory responses, resulting in neuron injury [39–41]. In the present study, we found that ROS production was increased in hypoxia-treated microglia (Figures 3(a) and 3(b)), whereas SOD and T-AOC activities were decreased (Figures 3(c) and 3(d)). In contrast, propofol was able to restore antioxidant activity (both SOD and T-AOC) in hypoxia-treated microglia (Figures 3(c) and 3(d)), thus inhibiting the production of ROS and inflammatory responses. It should be noted that the concentration of propofol was a key factor in this process and that high concentrations (100 *μ*M) were found to reduce antioxidant activity. This might be due to cytotoxicity at high concentrations [42, 43]. This decrease in antioxidant activity (both SOD and T-AOC) in hypoxia-treated microglia is consistent with previous studies [9, 44].

In addition, overproduction of ROS also results in the loss of mitochondrial membrane potential and aggravates mitochondrial dysfunction [8, 45, 46]. Furthermore, the effect of propofol on mitochondrial membrane potential and mitochondrial function is related to dose, cell type, and administration route [12, 47]. Studies have shown that propofol can prevent the collapse in membrane potential in liver and brain mitochondria during ischemia [11, 12]. In this study, we found that propofol could attenuate hypoxia-induced decreases in mitochondrial membrane potential (Figure 4) but failed to do so at 50 *μ*M concentration.

To explore the mechanism by which propofol affects hypoxia-induced inflammation in microglia, further experiments were carried out. NF-*κ*B is a key transcription factor that is associated with hypoxia-induced inflammation [29, 48–50]. Propofol can inhibit NF-*κ*B activation, thereby playing an anti-inflammatory role. In vivo, NF-*κ*B is a prerequisite for constitutive Hif-1*α* expression [17, 21]. At the same time, activated NF-*κ*B regulates Hif-1*α* expression and protects against thrombin, hydrogen peroxide, and even short-term hypoxia in vitro [15, 51]. To investigate the role of NF-*κ*B p65 in propofol-mediated protective effects, we knocked down NF-*κ*B p65 via siRNA transfection, followed by exposure to hypoxia for 24 h. We observed that hypoxia-induced Hif-1*α* and IL-1*β* production was downregulated with NF-*κ*B p65 silencing (Figures 5(d), 5(f), and 5(g)). In addition, pretreatment with propofol significantly inhibited the upregulation of NF-*κ*B p65 and Hif-1*α* production in BV2 microglia exposed to CoCl_2_ (Figures 5(a)–5(c)).

To summarize, our data show that propofol attenuates hypoxia-induced inflammation in BV2 microglia, in addition to reducing the production of ROS and enhancing antioxidant activity, at least in part by regulating the NF-*κ*B/Hif-1*α* signaling pathway. Further studies have to be performed to explore the effectiveness of propofol in clinical trials and to standardize the mode of administration with an appropriate dosage.

## Figures and Tables

**Table 1:** Primer sequence.

Primer	Sequence (from 5′ to 3′)
IL-6-F	CTCTGCAAGAGACTTCCATCC
IL-6-R	GAATTGCCATTGCACAACTC
iNOS-F	TTCACAGCTCATCCGGTACG
iNOS-R	CCATCAGCTTGCAAGACCAG
IL-1*β*-F	TAACCTGCTGGTGTGTGACG
IL-1*β*-R	TGTCGTTGCTTGGTTCTCCT
TNF-*α*-F	GTCCGGGCAGGTCTACTTTG
TNF-*α*-R	GGGGCTCTGAGGAGTAGACA
*β*-Actin-F	GGAGATTACTGCCCTGGCTCCTA
*β*-Actin-R	GACTCATCGTACTCCTGCTTGCTG

## Data Availability

The data used to support the findings of this study are available from the corresponding author upon request.

## Additional Points

*Highlights*. (1) Propofol reduces CoCl_2_-induced proinflammatory cytokine levels in BV2 cells. (2) Propofol alleviates CoCl_2_-induced oxidative stress and decrease in mitochondrial membrane potential. (3) Propofol attenuates hypoxia-induced neuroinflammation via inhibiting NF-*κ*B/Hif-1*α* signaling.
